# Effects of GLP-1 receptor agonist on changes in the gut bacterium and the underlying mechanisms

**DOI:** 10.1038/s41598-021-88612-x

**Published:** 2021-04-28

**Authors:** Shunsuke Kato, Takehiro Sato, Hiroki Fujita, Masahiro Kawatani, Yuichiro Yamada

**Affiliations:** 1 Departments of Endocrinology, Diabetes, and Geriatric Medicine, Akita University Graduate School of Medicine, Akita, Japan; 2 Departments of Neurophysiology, Akita University Graduate School of Medicine, Akita, Japan; 3 Department of Neuroscience II, Research Institute of Environmental Medicine, Nagoya University, Nagoya, Japan; 4 Kansai Electric Power Medical Research Institute, 2-1-7 Fukushima, Fukushima-ku, Osaka, Japan

**Keywords:** Endocrinology, Gastroenterology

## Abstract

There is a close relationship between the gut microbiota and metabolic disorders. In this study, acute administration of the glucagon-like peptide-1 receptor agonist (GLP-1RA) liraglutide to mice increased the cecal levels of caseinolytic protease B, a component of *Escherichia coli*, and of norepinephrine. Chemical sympathectomy blocked these events. Norepinephrine was found to pass into the intestinal lumen in vitro. c-Fos staining of the intermediolateral nucleus was identified as indirect evidence of sympathetic nervous system activation of the intestinal tract by GLP-1RA. Under normal conditions, the increase in *E. coli* did not affect the host. However, in mice with colitis, bacterial translocation was observed with attenuation of tight junction gene expression. This is the first study to investigate the unique underlying mechanisms related the effects of GLP-1RA on changes in the gut bacterium.

## Introduction

The gut microbiota exerts various effects on the host and is associated with several diseases^1^. For example, butyrate producing bacteria and *Akkermansia* are decreased in type 2 diabetes mellitus patients^2^. It has been reported that the intestinal environment (bacteria and viruses) changes even before the onset of type 1 diabetes mellitus (T1DM) and that there are bacteria that inhibit the onset of T1DM^3–5^. Meanwhile, anti-diabetic drugs have been reported to alter the structure of the gut microbiota. There are reports of beneficial changes in the gut microbiota by biguanides, but few other anti-diabetic drugs are reported^6–8^.

*Escherichia coli *(*E. coli*), one of the most famous bacteria of the gut microbiota, is a usually harmless resident bacterium. However, some *E. coli* strains can cause illness such as diarrhea^9^. An increase in *E. coli* has been reported in inflammatory bowel disease (IBD) patients^10^ and similar results have been found in a murine colitis model^11^. Certain types of *E. coli* have been reported to activate host intestinal defense factors and inhibit the growth of other harmful gut bacteria, and their roles are expected to be diverse^12,13^.

The effects of *E. coli* components on the host have recently been reported. Caseinolytic protease B (ClpB), a component protein of *E. coli*, suppresses host appetite^14^. An increase in lipopolysaccharide (LPS) after eating has also been found to help to improve postprandial hyperglycemia^15^. On the other hand, there are reports that LPS reduces tight junction gene levels and promotes bacterial translocation, and that amyloid release from *E. coli* is involved in autoimmune diseases such as T1DM and systemic lupus erythematosus^16–19^. Thus, fluctuations in the levels of *E. coli* can be both beneficial and detrimental to the host.

Various factors are involved in the fluctuations and stabilities in bacterial levels, such as nutrient availability, temperature, osmolality, pH, and bacteriophages, which are viruses that infect bacteria^20–23^. In addition, bacteria are regulated by host-derived neurotransmitters, hormones, and cytokines^20^. Norepinephrine (NE) has been reported to promote iron uptake in vitro, leading to the growth of *E. coli*^20^. Although NE is present in the intestinal lumen^24^, it is not clear whether NE in the intestinal tissue is released into the intestinal lumen and there appear to be no reports of NE-mediated increases in *E. coli *in vivo.

Gastrointestinal hormones that promote postprandial insulin secretion are collectively called incretins. There are two types: gastric inhibitory polypeptide (GIP), secreted from K cells present in the upper intestine, and glucagon-like peptide-1 (GLP-1), secreted from L cells present in the lower intestine and large intestine^25^. GLP-1 receptor agonists (GLP-1RAs) increase GLP-1 levels by 10 times or more and exert several pharmacological actions, including appetite-suppressant effects and delayed gastric emptying. The adverse effects of GLP-1RA, such as nausea, constipation, and diarrhea, are thought to be the result of the pharmacological action of GLP-1, but the mechanisms have not been fully elucidated. Nonetheless, GLP-1RA is known to affect the intestinal environment and, indeed, changes in the gut microbiota have been linked to GLP-1RAs^26–28^.

GLP-1RAs may directly or indirectly activate the sympathetic nervous system. One potential mechanism is thought to involve activation of the central nervous system, with the area postrema (AP) and nucleus of the solitary tract (NTS) and the intermediolateral nucleus (IML) of the spinal cord similarly activated by administration of GLP-1RA^29,30^. In general, GLP-1 is thought to have two effects, one exerted via the central nervous system and the other exerted via local receptors in the periphery. For example, GLP-1 may activate the heart, including the sinoatrial node, atrial cells, and ventricular cells, directly or indirectly through the sympathetic nervous system to increase the heart rate^31,32^. Similarly, two effects of GLP-1 on the gut must be borne in mind: sympathetic nervous system activation and direct effects on receptors in the gut tissue. In the latter, previous reports have shown that GLP-1R is involved in NO-mediated suppression of intestinal motility^33^. However, the relationship with intestinal NE and GLP-1 has not been clarified.

In this study, our hypothesis is that the increase in intestinal NE associated with sympathetic nervous system activation will play an important role in the association between the gut bacterium, especially *E. coli*, and GLP-1. We show the release of NE into the intestinal lumen in vitro and the activation of the sympathetic nervous system by acute administration of GLP-1RA with a concomitant rapid increase in *E. coli *in vivo. This is the first study to investigate the unique underlying mechanisms related the effects of GLP-1RA on changes in the gut bacterium.

## Results

### Acute administration of liraglutide increases E. coli levels in mice

**Figure 1:**
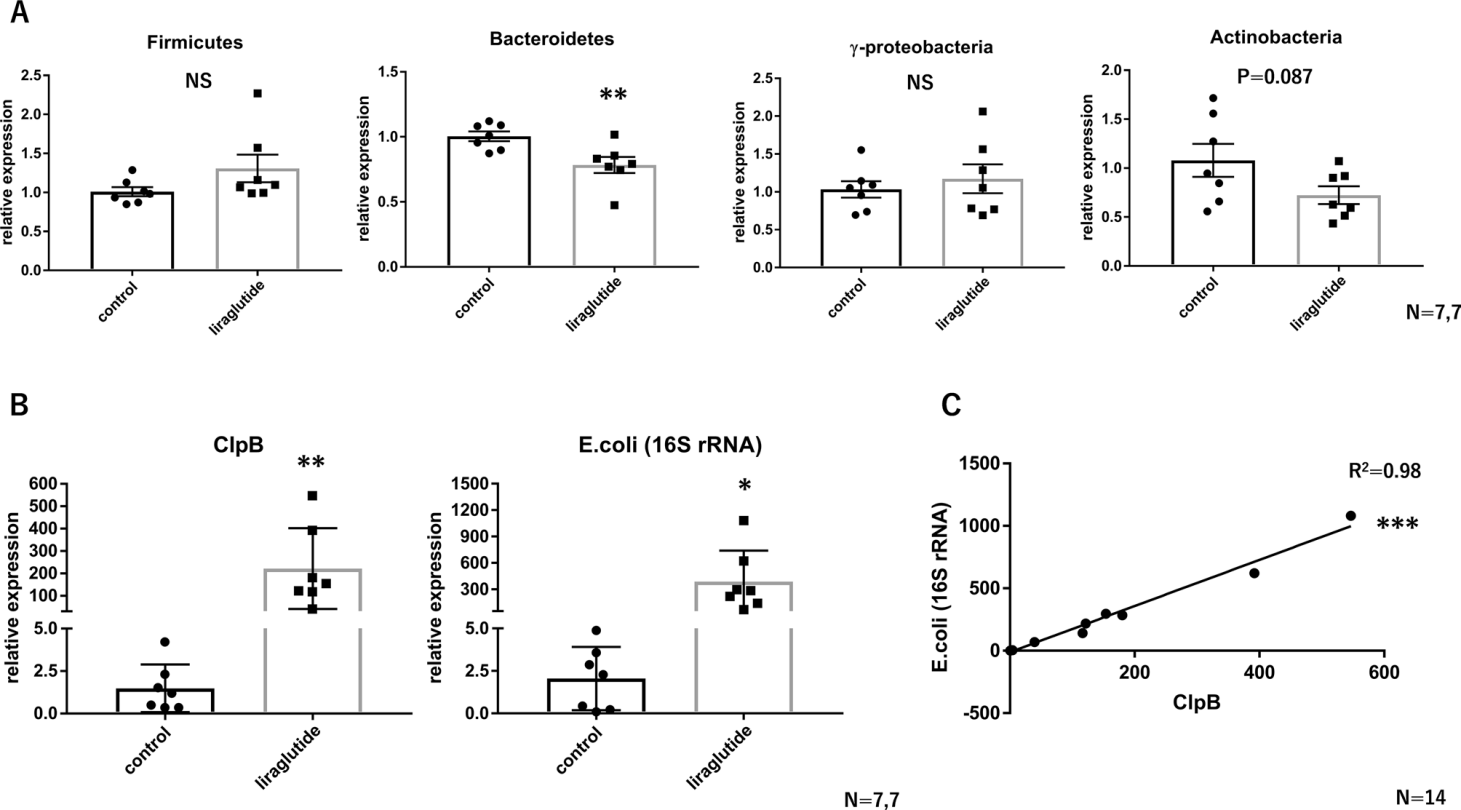
Acute administration of liraglutide increases *E. coli* levels in mice. Relative expression of (**A**) major gut microbiota (phylum level) and (**B**) ClpB and *E. coli* (16S rRNA) in cecal contents with or without acute liraglutide administration (control, n = 7; liraglutide, n = 7). (**C**) Correlation between the relative expression of ClpB and *E. coli* (16S rRNA) in cecal contents (n = 14). All values represent the mean ± SEM. *P < 0.05, **P < 0.01, ***P < 0.001; NS, not significant by non-paired t-test (**A** and **B**) or linear regression analysis (**C**). The coefficient of determination is denoted as R^2^. All figures were drawn using GraphPad Prism 7 (www.graphpad.com) and Microsoft’s PowerPoint 2016 (www.microsoft.com).

After a single subcutaneous injection of liraglutide, a GLP-1RA, followed by a 16-h overnight fast, the relative expression levels of the gut bacterium in the cecal contents of mice were assessed using quantitative PCR. At the phylum level, liraglutide administration significantly decreased *Bacteroidetes* and tended to increase *Actinobacteria* (Fig. 1A). However, *Firmicutes* and *Proteobacteria* were not changed (Fig. 1A). At the genus level, liraglutide administration significantly reduced *Ruminococcus* and did not increase *Akkermansia* (Supplementary Fig. 1A).

We then compared the gene expression levels of bacterial proteins that could affect the host metabolism. Expression of formate–tetrahydrofolate ligase (FTHFS), which is related to acetic acid synthesis, was significantly increased and, conversely, the expression of butyryl-CoA: acetate CoA-transferase (BCoAT), which is related to butyrate synthesis, was significantly decreased (Supplementary Fig. 1A). We also showed that acute administration of liraglutide increased the expression of ClpB by about 400-fold compared with the control (Fig. 1B). ClpB is encoded by several bacteria, but it is the ClpB from *E. coli* that affects host metabolism. To determine whether the expression of ClpB is truly related to *E. coli* number*,* we examined the 16S rRNA expression of *E. coli* and identified a significant positive correlation with ClpB (Fig. 1C). Thus, we have shown that acute administration of liraglutide increases *E. coli* levels in mice.

We also performed the experiment in the non-fasting condition. In the non-fasting condition, the expression of ClpB in the cecum contents was significantly higher in the liraglutide group. (Supplementary Fig. 1B). However, since liraglutide administration significantly reduced food intake (Supplementary Fig. 1C), we chose the fasting condition for this study. Also, in the present study, we focused in subsequent experiments on the dramatic elevation in ClpB, as a marker of *E. coli*, induced by liraglutide administration.

### Liraglutide increases NE levels in cecal contents via activation of sympathetic nerves

**Figure 2:**
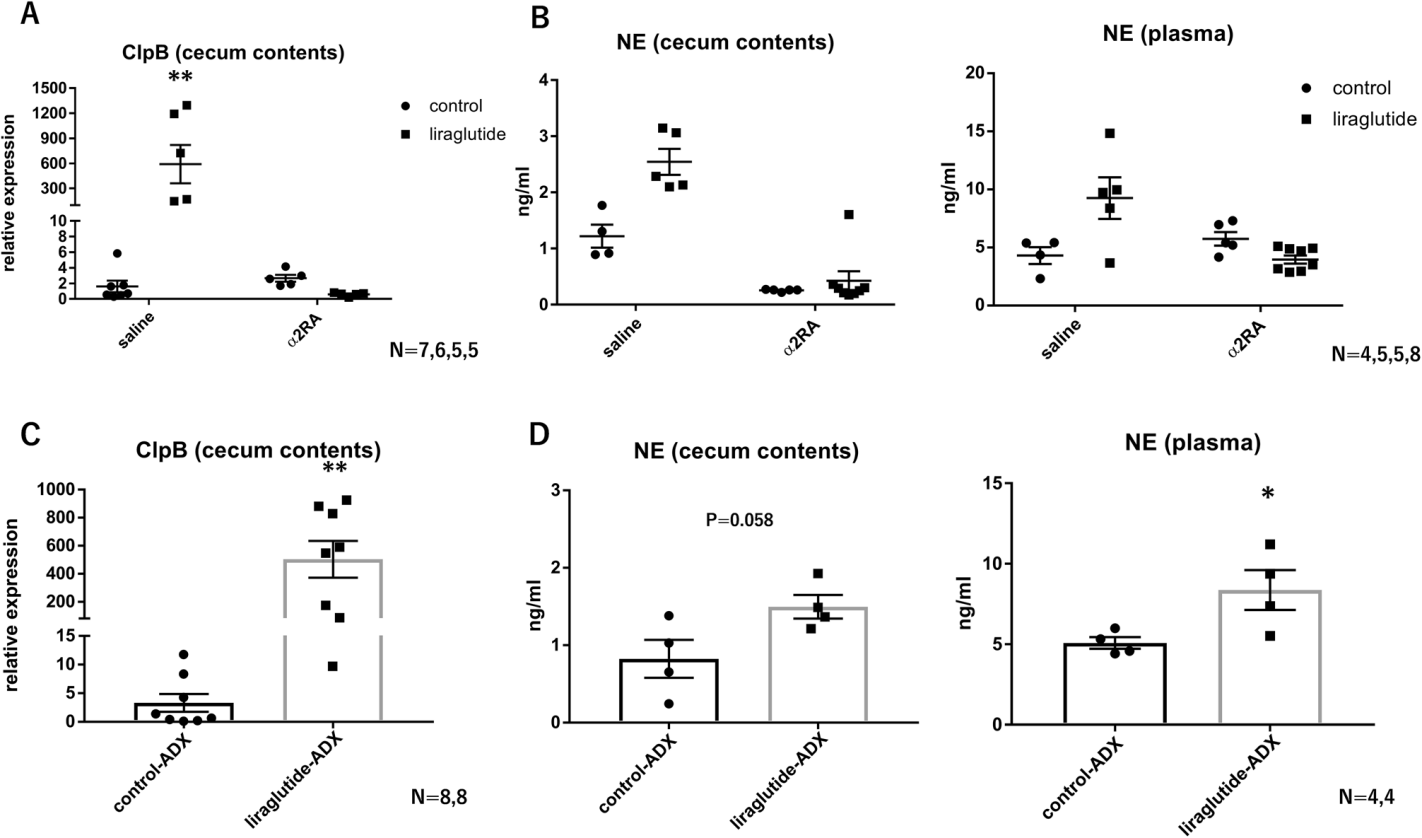
Liraglutide increases NE levels in cecal contents via activation of sympathetic nerves. (**A**) Relative expression of ClpB in cecal contents in saline and medetomidine (α2 receptor agonist [α2RA]) groups with or without acute liraglutide administration (saline control, n = 7; saline liraglutide, n = 6; α2RA control, n = 5; α2RA liraglutide, n = 5). (**B**) Measurement of norepinephrine concentrations in cecal contents and plasma in saline and medetomidine groups with or without acute liraglutide administration (saline control, n = 4; saline liraglutide, n = 5; α2RA control, n = 5; α2RA liraglutide, n = 8). (**C**) Relative expression of ClpB in cecal contents with or without acute liraglutide administration under bilateral adrenalectomy (ADX) (control-ADX, n = 8; liraglutide-ADX, n = 8). (**D**) Measurement of norepinephrine concentrations in cecal contents and plasma with or without acute liraglutide administration under ADX (control-ADX, n = 4; liraglutide-ADX, n = 4). All values represent the mean ± SEM. *P < 0.05, **P < 0.01, ***P < 0.001; NS, not significant by two-way ANOVA with multiple comparison test (**A** and **B**) or non-paired t-test (**C** and **D**). All figures were drawn using GraphPad Prism 7 (www.graphpad.com) and Microsoft’s PowerPoint 2016 (www.microsoft.com).

Next, we investigated whether sympathetic nervous system activation was involved in the increase in *E. coli* by examing levels of intestinal NE. We showed that acute administration of liraglutide significantly increased NE in both plasma and cecal contents (Fig. 2A). Then, administration of liraglutide with medetomidine, an α2 receptor agonist, which suppresses the sympathetic nervous system, did not increase ClpB expression in cecal contents and NE levels in plasma and cecal contents (Fig. 2A,B). When liraglutide was acutely administered to bilaterally-adrenalectomized mice, NE levels in cecal contents tended to be increased and the expression of ClpB in cecal contents and NE levels in plasma were significantly increased (Fig. 2C,D). These results indicated that NE in cecal contents could be derived from sympathetic nerve terminals.

### NE from sympathetic nerves passes into the intestinal lumen

**Figure 3:**
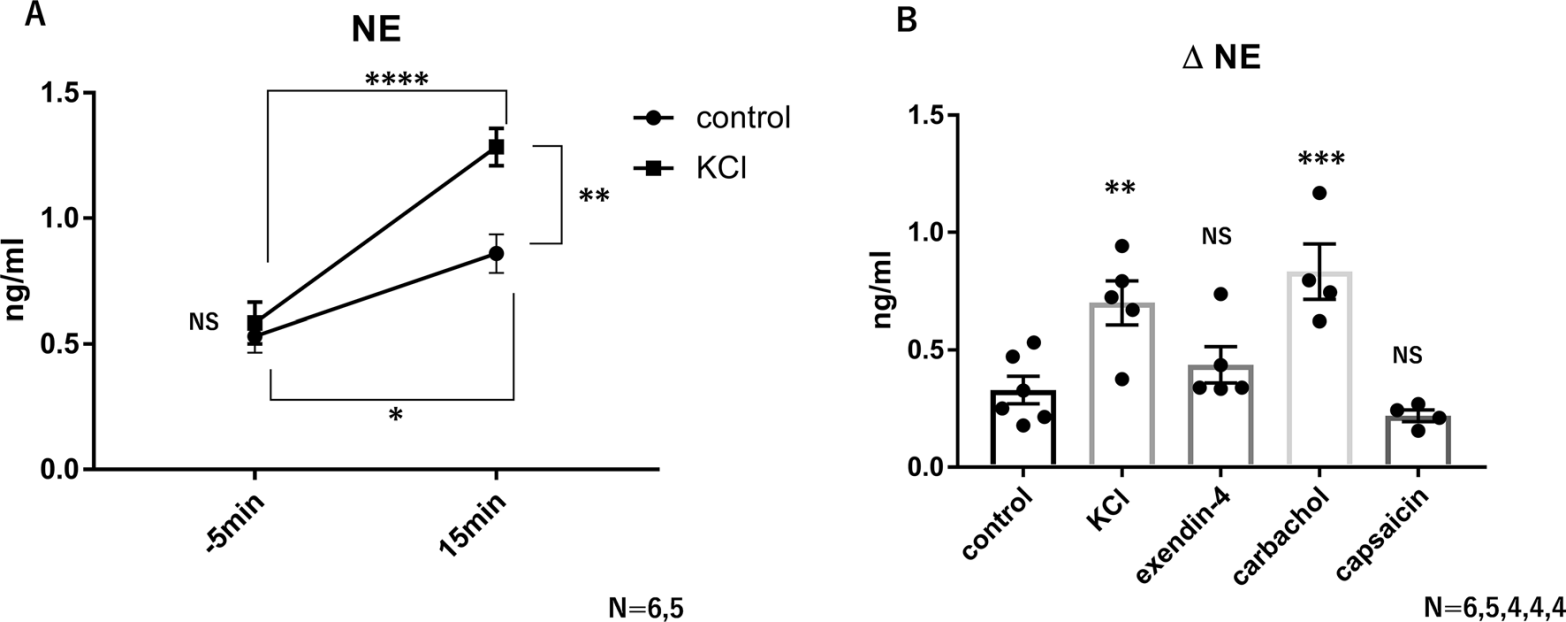
NE from sympathetic nerves passes into the intestinal lumen. (**A**) Measurement of norepinephrine concentration on the mucosal side before and after KCl administration to the serosal side of the cecum (control, n = 6; KCl, n = 5). (**B**) Change in norepinephrine concentration on the mucosal side after the administration of various drugs to the serosal side of the cecum (control, n = 6; KCl, n = 5; exendin-4, n = 4; carbachol, n = 4; capsaicin, n = 4). All values represent the mean ± SEM. *P < 0.05, **P < 0.01, ***P < 0.001; NS, not significant by two-way ANOVA with multiple comparison (**A**) or one-way ANOVA with multiple comparison (**B**). All figures were drawn using GraphPad Prism 7 (www.graphpad.com) and Microsoft’s PowerPoint 2016 (www.microsoft.com).

We used a Ussing chamber to investigate whether the NE concentration on the mucosal side of the cecal tissue was increased by administration of drugs to the serosal side. A high concentration of KCl (60 mM) was administered to depolarize the cells, including the sympathetic nerve endings, and an increase in the NE concentration on the mucosal side was confirmed (Fig. 3A,B). We have recently reported the effect of carbachol on nicotinic receptors, and since nicotinic receptors are expressed at sympathetic synapses, we administered carbachol^34^. As a result, we found that carbachol administration increased the NE concentration. (Fig. 3B). GLP-1 receptors are expressed in the afferent branch of the vagus nerve and the enteric nervous system (ENS). However, activation of the afferent branch of the vagus nerve by capsaicin or activation of the vagus nerve and ENS by exendin-4, another GLP-1RA, failed to increase NE concentrations (Fig. 3B). These results indicate that NE in the intestinal tissue passes into the intestinal lumen and that GLP-1 acts more centrally.

### Liraglutide enhances c-Fos staining, not only in the AP/NTS, but also in the IML

**Figure 4:**
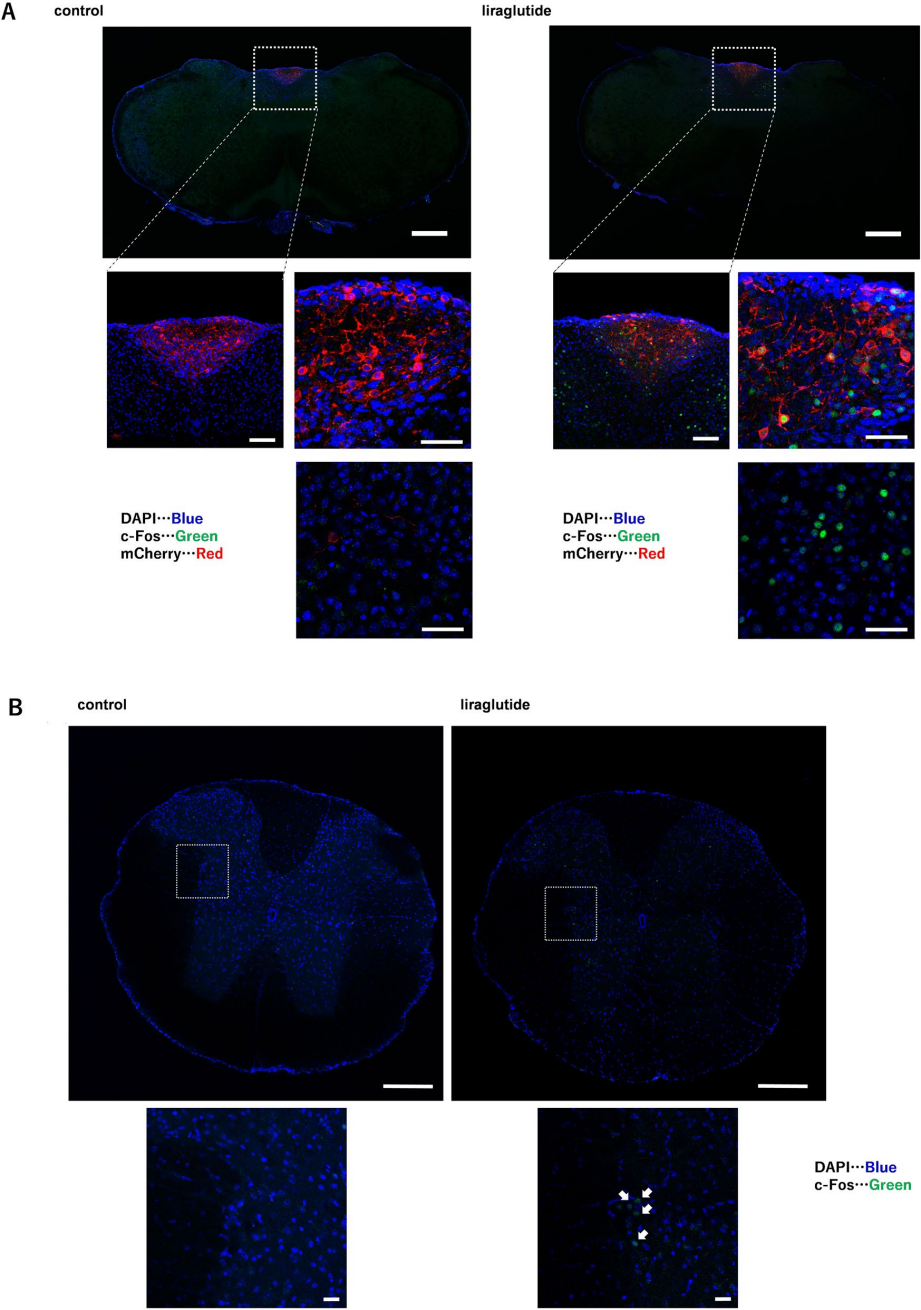
Liraglutide enhances c-Fos staining, not only in the AP/NTS, but also in the IML. Representative image of immunohistochemistry for c-Fos in (**A**) the AP and NTS and (**B**) the IML with or without acute liraglutide administration. In (**A**), the top and middle left images are the original magnification, whereas the middle right and lower images are the original magnification × 40. Scale bars: 500 µm in the top image, 100 µm in the middle left image, and 50 µm in the middle right and lower images in (**A**). All images are the original magnification in (**B**). Scale bars: 200 µm in the top image, 20 µm in the lower image in (**B**). Arrows indicate c-Fos staining in (**B**).

Because no direct peripheral effects of GLP-1RA were observed, we next examined its effects on the central nervous system. The brainstem nuclei, followed by preganglionic neurons in the IML, transmit postganglionic signals to the intestine. Two hours after liraglutide administration, c-Fos staining was clearly observed in the AP and NTS (Fig. 4A) and in the IML (Fig. 4B), suggesting that one mechanism by which GLP-1RA activates the sympathetic nervous system is a pathway involving the activation of the IML via the AP and NTS.

### Increased expression of ClpB does not affect appetite

We next investigated the effects of a marked increase in *E. coli* in the gut on the host. ClpB has been reported to suppress appetite^14,35^. Liraglutide was administered to mice after suppression of the gut microbiota by antibiotics. The results showed that liraglutide had a significant appetite-suppressant effect, even in antibiotic-treated mice (Supplementary Fig. 2A,B). Next, the effect of appetite suppression was confirmed via fecal microbiota transplantation (FMT). Mice administered a single dose of liraglutide were used as donors, and antibiotic-treated mice were used as recipients. Recipient food intake during refeeding was then measured, but no significant difference was observed between the control and liraglutide-treated groups (Supplementary Fig. 2C). Therefore, the association between an increased expression of ClpB and appetite suppression after administration of liraglutide was considered to be weak.

### In dextran sulfate sodium colitis, liraglutide attenuates tight junction gene expression in the cecum

**Figure 5:**
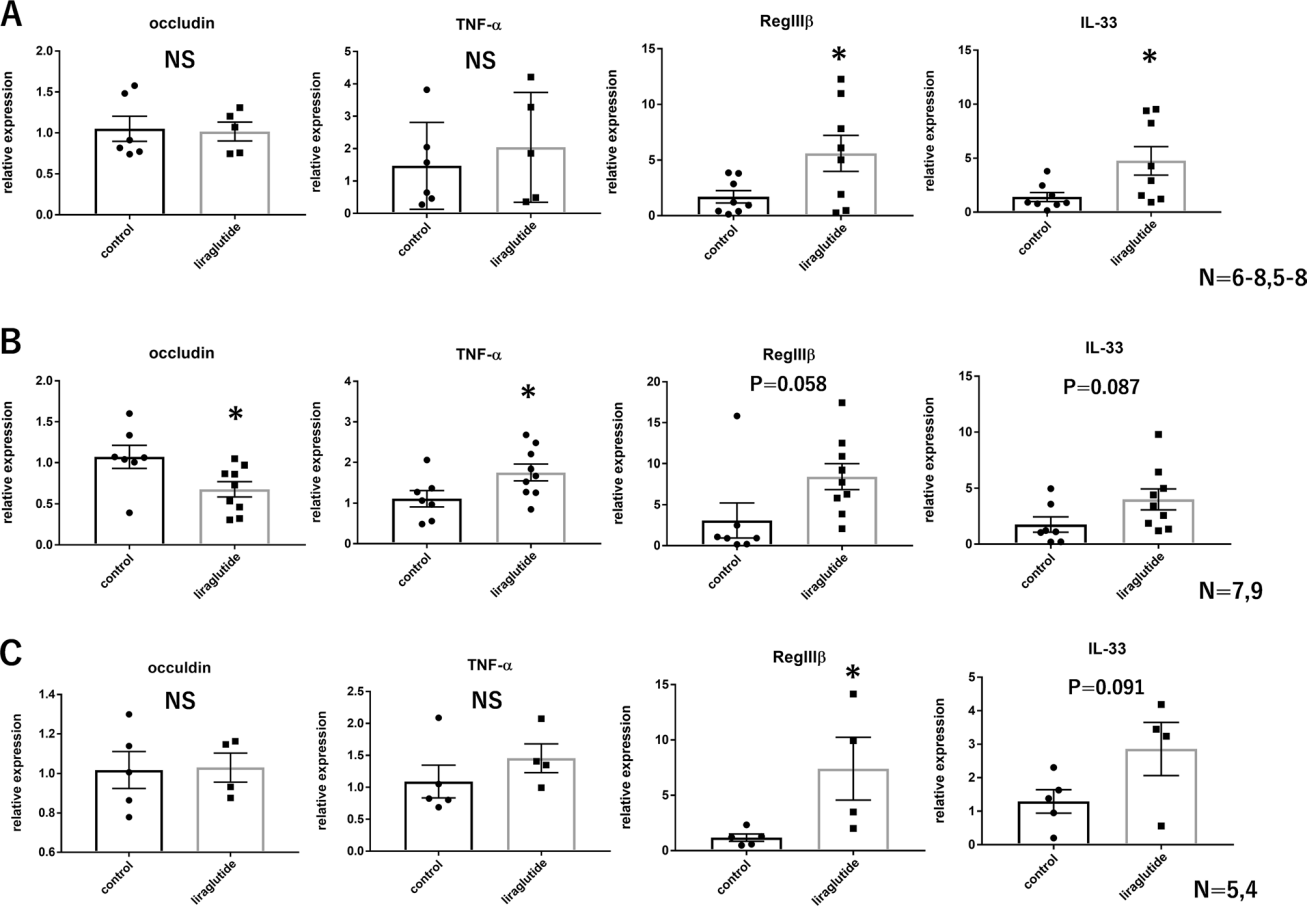
In DSS colitis, liraglutide attenuates tight junction gene expression in the cecum. Relative expression of cecal genes with or without acute liraglutide administration (**A**) in wild-type mice (control, n = 6–8; liraglutide, n = 5–8), (**B**) in wild-type mice (control, n = 7; liraglutide, n = 9), and (**C**) in TLR4KO mice with DSS colitis (control, n = 5; liraglutide, n = 4). All values represent the mean ± SEM. *P < 0.05, **P < 0.01, ***P < 0.001; NS, not significant by non-paired t-test (**A**–**C**). All figures were drawn using GraphPad Prism 7 (www.graphpad.com) and Microsoft’s PowerPoint 2016 (www.microsoft.com).

Given that LPS, a component of Gram-negative bacteria such as *E. coli*, is reported to reduce the levels of intestinal tight junction mRNA^16,17^, we examined the effects of liraglutide administration on intestinal barrier function. Under normal conditions, acute administration of liraglutide did not alter cecal occludin and TNF-α mRNA levels, but significantly increased cecal RegIIIβ and IL-33 mRNA levels (Fig. 5A). In the dextran sulfate sodium (DSS) colitis model, the liraglutide group had significantly lower cecal occludin mRNA levels and significantly higher TNF-α mRNA levels compared with the control group, with no difference in body weight (Fig. 5B, Supplementary Fig. 3A). Reg IIIβ and IL-33 mRNA levels tended to be higher in the liraglutide group than in the control group (Fig. 5B). There was no difference between the two groups in the levels of other genes involved in intestinal barrier function (Supplementary Fig. 4). Because toll-like receptor-4 (TLR4) is a receptor for LPS, subsequent experiments were conducted using TLR4 knockout (TLR4KO) mice. Acute administration of liraglutide to TLR4KO mice during DSS colitis increased the expression of ClpB in cecal contents, consistent with the results in wild-type mice (Supplementary Fig. 3C). We found that the mRNA levels of cecal occludin and TNF-α were not different between the liraglutide group and the control group, with no difference in body weight (Fig. 5C, Supplementary Fig. 3B), suggesting that the liraglutide-induced reduction in occludin gene expression levels was LPS- and TLR4-dependent. In addition, cecal RegIIIβ and IL-33 mRNA levels were significantly higher or tended to be higher than in the control group (Fig. 5C), suggesting that the increase in the levels of these genes was LPS- and TLR4-independent.

### In DSS colitis, liraglutide promotes bacterial translocation

**Figure 6:**
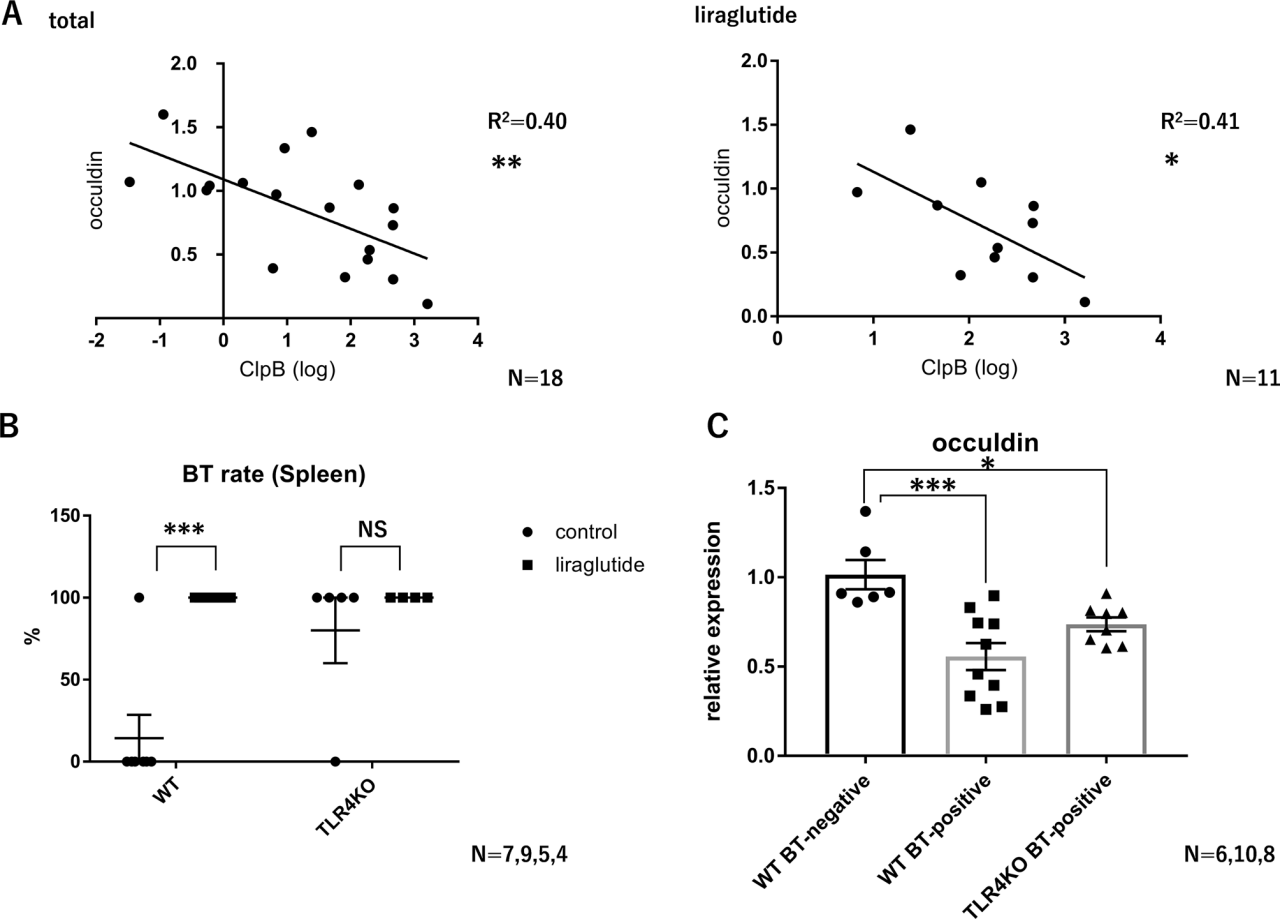
In DSS colitis, liraglutide promotes bacterial translocation. (**A**) Correlation between the relative expression of ClpB in cecal contents and occludin in cecal tissue in mice with DSS colitis with or without acute liraglutide administration (total, n = 18; liraglutide, n = 11). (**B**) ClpB positive rate in the spleen under DSS colitis conditions in wild-type (WT) mice and TLR4KO mice with or without acute liraglutide administration (WT control, n = 7; WT liraglutide, n = 9; TLR4KO control, n = 5; TLR4KO liraglutide, n = 4). (**C**) Cecal occludin gene expressions according to ClpB positivity (bacterial translocation [BT]) under DSS colitis conditions in wild-type (WT) mice and TLR4KO mice (WT BT-positive, n = 6; WT BT-negative, n = 10; TLR4KO BT-positive, n = 8). All values represent the mean ± SEM. *P < 0.05, **P < 0.01, ***P < 0.001; NS, not significant by linear regression analysis (**A**), two-way ANOVA with multiple comparison test (**B**), and one-way ANOVA with multiple comparison test (**C**). The coefficient of determination is denoted as R^2^. All figures were drawn using GraphPad Prism 7 (www.graphpad.com) and Microsoft’s PowerPoint 2016 (www.microsoft.com).

Cecal occludin mRNA levels showed a significant negative correlation with the relative expression of ClpB in cecal contents (Fig. 6A). To examine intestinal barrier function, we investigated the rate of bacterial translocation (BT) using spleen DNA. The positive rate was 100% (9 of 9) in the liraglutide group but just 14.3% (1 of 7) in the control group (Fig. 6B). In TLR4KO mice, the positive rates of BT were 100% (4 of 4) in the liraglutide group and 80% (4 of 5) in the control group (Fig. 6B). Cecal occludin mRNA levels were significantly lower in the BT-positive group than in the BT-negative group (Fig. 6C). Occludin mRNA levels were significantly lower in the BT-positive group of TLR4KO mice than in the BT-negative group of wild-type mice (Fig. 6C). Accordingly, acute administration of liraglutide may attenuate intestinal tight junction gene levels via an LPS- and TLR4-dependent mechanism and promote BT under stress conditions, such as those found in the colitis model.

## Discussion

There are several reports on the effects of GLP-1RA on the gut microbiota in rodents^26–28^. However, the changes in the gut microbiota were not completely consistent among these studies. In addition, none of the studies discussed the changes in *E. coli* levels with GLP-1RA administration^26–28^. These reports were based on the study of gut microbiota in the feces after chronic administration of GLP-1RA. In the present work, liraglutide was acutely administered and DNA was extracted from cecal content. The gut microbiota in the cecum and feces are similar, but not necessarily the same. For example, more *E. coli* is detected in the cecum than in the feces^36,37^. Next, considering the duration of drug administration, GLP-1RA has a diminished effect after chronic administration, also known as tachyphylaxis, although the mechanism of tachyphylaxis is currently unclear. Liraglutide is a long-acting GLP-1RA that is more prone to tachyphylaxis related to delayed gastric emptying than short-acting GLP-1RAs such as exenatide^38^. Therefore, the changes in the gut microbiota after a single injection of GLP-1RA seem to reflect the original effects of GLP-1RA more clearly than the changes in the gut microbiota after chronic administration. The present study is significant in that it is the first to reveal a rapid increase in *E. coli* with acute administration of GLP-1RA.

We have shown that liraglutide administration increases NE levels in plasma and cecal contents. Asano et al. have already reported the presence of NE in the intestinal tract^24^. They showed that intestinal NE was reduced but still present in germ-free mice^24^. In other words, intestinal NE could be derived from two sources: the gut microbiota and the host. Here, we discovered that some of the NE in cecal contents is derived from the sympathetic nerves of the host because the liraglutide-induced NE increase was unaffected by adrenalectomy but suppressed by chemical sympathetic nerve blockade. Indeed, via the Ussing chamber, we confirmed that NE can pass into the intestinal lumen. Our results strongly suggest that GLP-1RA causes a surge of *E. coli* due to sympathetic nervous activation and subsequent increased NE release into the intestinal lumen.

Next, we must discuss GLP-RA and sympathetic nervous system activation. Two mechanisms of intestinal sympathetic nervous system activation by GLP-1RA must be considered: central nervous system-mediated effects and direct peripheral effects. The ENS also expresses GLP-1 receptors^39^, but the local effects of GLP-1 could not be proven because GLP-1RA was ineffective in our studies in Ussing chamber experiments. In the present study, subcutaneous administration of liraglutide led to activation in the NTS, AP, and IML. The result suggests that sympathetic nervous system activation by GLP-1RA is due to direct activation of AP without blood–brain barrier or indirect activation of NTS via vagal afferent nerves and AP. Indeed, we have reported that GLP-1 receptors are expressed in NTS and AP neurons and are depolarized by administration of GLP-1^30^. Recently, it has been reported intraperitoneal administration of GLP-1RA activates mesenteric ganglia located periphery to the IML and inhibits intestinal motility^40^,which is consistent with our results. The present study is significant because it shows that the increase in intestinal NE caused by GLP-1RA is due to the involvement of central nervous system activation in addition to potential peripheral effects of GLP-1.

We need to also discuss the risks of an increase in *E. coli*. First, we must consider the adverse abdominal effects of GLP-1RA, which include nausea, abdominal distention, constipation, and diarrhea^41,42^. Nausea may be due to delayed gastric emptying, and abdominal distention and constipation may be due to delayed intestinal motility, but the reason for the diarrhea is unclear. Some *E. coli* strains can cause diarrhea. Therefore, the possibility that an increase in *E. coli* can cause diarrhea cannot be ruled out. We observed no diarrhea after acute administration of liraglutide. Investigation of the causal relationship between the increase in *E. coli* and diarrhea caused by GLP-1RA use is an issue for future study. Secondly, we examined the BT of *E. coli.* Under DSS colitis conditions, we observed that liraglutide administration attenuated occludin mRNA levels and increased TNF-α mRNA levels in the cecum. Occludin gene expression was negatively correlated with ClpB expression in cecal contents, suggesting that occludin may have been affected by *E. coli*. There is no report that GLP-1RA increases BT, but there are reports that GLP-1 levels are high in critically ill patients and those with sepsis^43,44^. Furthermore, elevated levels of GLP-1 have been reported in patients with IBD^45,46^. In addition, the severity of the condition has been associated with an increase in *E. coli* in a murine IBD model^11^. However, there has been no concurrent investigation of the increase in GLP-1, *E. coli*, and IBD severity, and a future study in humans should be conducted.

Additional important aspects are the benefits of increased levels of *E. coli* and the new significance of GLP-1. To begin with, GLP-1 is secreted by L cells in the lower intestine, and its secretory stimuli include nutrients, the gut microbiota itself, and metabolites of the gut microbiota, such as short-chain fatty acids^47,48^. These stimuli are foreign substances and the purpose of the sympathetic nervous system activation may be to protect the host from foreign substances. Indeed, activation of the sympathetic nervous system activates immune cells, which have also been reported to be potentially beneficial to the host^49^. Thus, are changes in the gut bacterium itself beneficial to the host? Reports that increased postprandial *E. coli* suppresses postprandial blood glucose or appetite in the host may be the result of interactions between the host and the gut microbiota^14, 15^. In other words, the gut microbiota and the host may have evolved with each other to produce mutually beneficial outcomes. In the present study, the effect of the increased ClpB on appetite is not clear, and inhibition of glycogenesis by increased LPS has not been examined. In terms of the host–bacterial symbiosis, changes in the gut microbiota may benefit the host and should be examined in detail in the future.

The limitations of our study are as follows. First, this study was limited to a single dose and fasting conditions because we excluded the effect of diet and examined the direct effect of GLP-1RA. Another approach would have been to conduct a pair-feeding study, but this was not done because it was not possible to make all feeding conditions exactly the same. In addition, it is difficult to apply the results of this study to humans because it is not common to administer GLP-1RA under fasting conditions in humans. Secondly, this study was limited to changes in *E. coli.* Although metagenomic analysis using Next-Generation Sequencing (NGS) is useful for estimating the overall view of the gut microbiota, it is less sensitive than qPCR at the species or strain level^50^. It has been reported that the data obtained by NGS and qPCR are correlated, so the results of this study will not be changed by NGS^51^. However, the analysis of the gut microbiota using NGS after a single administration of GLP-1RA has not been studied, and this is a subject for further study. Third, we may not be controlling the TLR4 ligands, since there are many TLR4 ligands other than LPS. For example, GLP-1RA has been reported to alter free fatty acid and LDL cholesterol levels which are TLR4 ligands^52,53^. Therefore, it is necessary to consider the possibility that these changes affected the results. Finally, with regard to BT, the viability of the bacteria is unclear, and the translocation of bacteria other than *E. coli* has not been examined. For the former, BT has been defined as the migration of bacteria or bacterial products from intestinal contents to extra-intestinal organs^54^. In several previous papers, BT has been confirmed by using PCR, therefore we also confirmed the BT of *E. coli* by using PCR^55–57^. However, the viability of the bacteria is unknown, and this is a subject for further study. In the latter, *E. coli* has been reported to be one of the easiest bacteria to translocate^58^. It has also been reported that certain *E. coli* are suitable for determining BT^59^. Therefore, we selected *E. coli* as the indicator of BT. BT of other bacteria is also a future issue.

**Figure 7:**
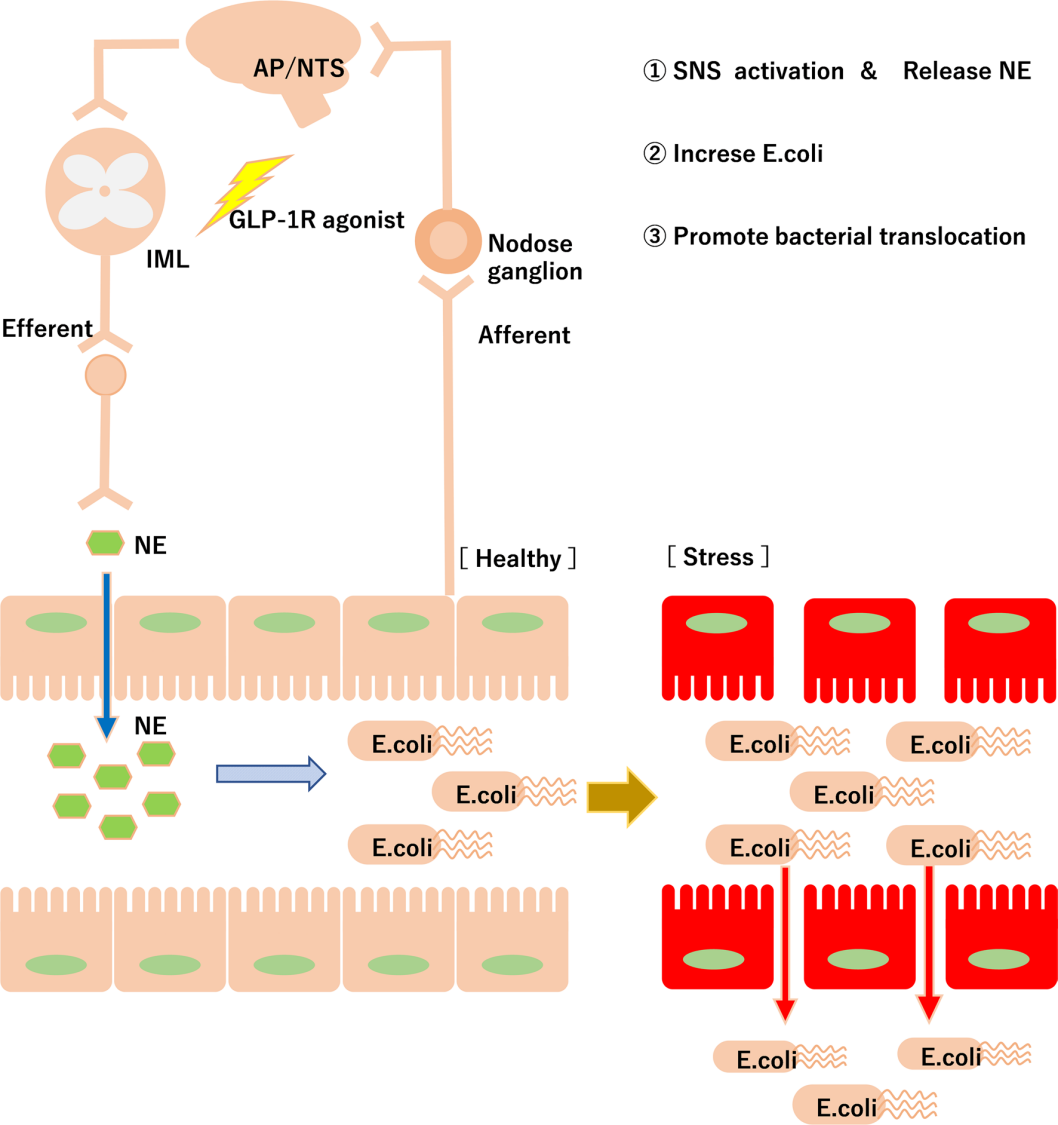
Schematic of this experiment. Acute administration of liraglutide causes these three phenomena: (1) sympathetic nervous system (SNS) activation and release of norepinephrine to the intestinal lumen, (2) increased levels of *E. coli* in cecal contents, and (3) promotion of bacterial translocation under stress conditions, such as colitis. This image was drawn using Microsoft’s PowerPoint 2016 (www.microsoft.com).

In conclusion, we show for the first time that one mechanism for the GLP-1RA-induced increase in intestinal *E. coli* is related to sympathetic nervous system activation and the subsequent release of NE into the intestinal lumen (Fig. 7). The increase in *E. coli* may help to promote BT by attenuating intestinal tight junction gene levels under stress conditions (Fig. 7). Finally, the benefits of increased levels of *E. coli* are a subject for further study.

## Materials and methods

### Mouse experiments

C57BL/6J mice were used at 8–16 weeks of age. Mice were housed in a controlled environment under a 12-h light/dark cycle and with ad libitum access to standard rodent chow and water. Animal experiments were carried out in accordance with the animal welfare guidelines of Akita University and the ARRIVE (Animal Research: Reporting of In Vivo Experiments) guidelines. All procedures were approved by the committee on animal experimentation of Akita University. We housed each mouse individually in a cage. Mice were subcutaneously injected with liraglutide (200 µg/kg body weight) (NOVO Nordisk, Bagsvaerd, Denmark) or saline. The relative ClpB expression levels at 2, 6, and 16 h after liraglutide administration were examined (Supplementary Fig. 5), and based on the results, “16 h” was adopted for this study. After overnight fasting for 16 h, we collected blood from the inferior vena cava of mice anesthetized with isoflurane. The contents of the cecum were collected. The cecal tissue was immediately frozen with dry ice and stored at − 80 °C. The same experiment was also performed in the non-fasting condition.

### Chemical sympathectomy study

Intraperitoneal administration of an α2-adrenoreceptor agonist (medetomidine 600 μg/kg body weight; Nippon Zenyaku Kogyo Co., Ltd., Tokyo, Japan) was used for chemical sympathectomy. The control group received the same amount of intraperitoneal saline. Subsequently, dissections were performed under the same conditions as above.

### Adrenalectomy study

A combination anesthetic was prepared with medetomidine (0.3 mg/kg body weight; Nippon Zenyaku Kogyo Co., Ltd.), midazolam (4 mg/kg body weight; Astellas Pharma Inc., Tokyo, Japan), and butorphanol (5 mg/kg body weight; Meiji Seika Pharma Co., Ltd., Tokyo, Japan). The median back skin and the back muscles were incised longitudinally at two locations on the left and right sides to expose the adrenal glands and were resected with scissors. After completion of the operation, 1 ml of warmed saline and dexamethasone (1 mg/kg body weight; FUJIFILM Wako Pure Chemical Corporation, Osaka, Japan) were intraperitoneally administered, and atipamezole (2.5 mg/kg body weight; Nippon Zenyaku Kogyo Co.) was also intraperitoneally administered for reversal. Thereafter, 0.9% saline was supplied for drinking. Four weeks later, liraglutide or saline was injected subcutaneously and dissection was performed as above.

### Antibiotics study

Antibiotics (ampicillin 1 g/L, neomycin 1 g/L, metronidazole 1 g/L; FUJIFILM Wako Pure Chemical Corporation) were administered in drinking water to depress gut microbiota. Two weeks after initiation of the antibiotics, we administered a single subcutaneous injection of liraglutide or saline to the mice. After the mice were fasted for 16 h, food intake was measured 1, 3, 6, and 24 h later. Mice that drank water without antibiotics were used as controls to perform experiments in the same way.

### Fecal microbiota transplantation study

Donor mice were subcutaneously injected with liraglutide or saline under fasting conditions and cecal contents were collected the following morning under isoflurane anesthesia. With 0.25 g of cecal contents per 1 ml of sterilized water, the prepared liquid was passed through a 70-µm cell strainer. The recipient mice were fed water with antibiotics for 2 weeks and changed to normal water 2 days before the fecal microbiota transplantation (FMT) was performed. After the recipient mice were fasted overnight for 16 h, the prepared liquid was administered orally and food intake was measured after 1, 3, 6, and 24 h.

### Dextran sulfate sodium colitis study

Wild-type mice (8–12 weeks of age) were allowed ad libitum access to drinking water containing 1.5%–2% dextran sulfate sodium (DSS, MW = 36,000–50,000; MP Biomedicals, Solon, OH) for 8–12 days (4–5 animals/cage). In addition, toll-like receptor-4 knockout (TLR4KO) mice (10–12 weeks of age; Oriental Bio Service, Inc., Kyoto, Japan) were allowed ad libitum access to drinking water containing 1.5% DSS for 8 days. Body weight was measured every 2 to 3 days. One day before dissection, body weight measured and the DSS-containing water was changed to normal water. After administration of liraglutide or saline, the mice were fasted for 16 h and the dissection was performed as above.

### Ussing chamber study

Nonfasted mice were dissected under isoflurane anesthesia, and the cecal tissue was removed and washed well with Krebs Ringer solution (117 mM NaCl, 5.9 mM KCl, 2.5 mM CaCl_2_, 1.2 mM MgCl_2_, 24.8 mM NaHCO_3_, 1.2 mM NaH_2_PO_4_, and 11.1 mM glucose). The opened cecal tissue was mounted to act as a 19.6-mm^2^ diaphragm between the two halves of a customized Ussing chamber. The mucosal side of the chamber had a volume of 1 ml and the serosal side had a volume of 500 µl. The chambers were filled with Krebs Ringer solution with bubbling 95% O_2_-5% CO_2_. In the chemical stimulation assay, KCl (60 mM), carbachol (100 µM; Sigma-Aldrich, St. Louis, MO), exendin-4 (100 nM; Sigma-Aldrich), and capsaicin (10 µM; Sigma-Aldrich) were added to the serosal side for 15 min. Then, 50 µl of samples were taken from the mucosal side 5 min before and 15 min after drug administration. The NE concentration in the sample was measured using the Noradrenaline Research ELISA kit (BA-E-5200; LDN, Nordhorn, Germany).

### c-Fos staining

We intraperitoneally injected the adeno-associated virus (AAV) series (AAV-CAG-GFP, Addgene, MA, USA) into neonatal mice and found that the AP region was labeled with AAV-9. Therefore, in the c-Fos staining of the AP and NTS, we used mice injected with AAV-9-CaMKIIa-hChR2(E123T/T159C)-mCherry (3 × 10e13, 2 μl) (Penn Vector Core, Philadelphia, PA) during the neonatal period. After 4 h of fasting, 8–12-week-old wild-type mice were subcutaneously injected with liraglutide (400 µg/kg body weight) or saline and, 2 h later, were dissected under inhalation anesthesia with isoflurane. The mice were intracardially perfused with phosphate-buffered saline (PBS) followed by cold 4% paraformaldehyde (PFA) in PBS. Immediately after fixation, the brain and spinal cord were manually removed. They were embedded in agarose, and a specimen with a cross-section thickness of 80 µm was prepared. After three washes with PBS, the sections were pre-incubated in blocking solution (5% serum albumin, 2% normal goat serum, and 0.3% Triton X-100 in PBS) for 20 min at room temperature, then incubated with an anti-c-Fos rabbit antibody (#sc-52, 1:2000; Santa Cruz Biotechnology, Santa Cruz, CA) and an anti-mCherry rat antibody (#M11217, 1:1000; Life Sciences, Madison, WI) in blocking solution for 16 h at 4 °C. After three washes with PBS, the sections were incubated with goat anti-rabbit IgG antibody coupled with Alexa Fluor 488 (1: 500; Invitrogen, Carlsbad, CA) and donkey anti-rat IgG antibody coupled with Cy3 (1: 250; Jackson ImmunoResearch Inc., West Grove, PA) in blocking solution for 90 min at room temperature. After three washes with PBS, the sections were coverslipped using Vectashield mounting medium with DAPI (Vector, Burlingame, CA). Fluorescent images were obtained using confocal laser microscopy (LSM510; Carl Zeiss, Jena, Germany). For spinal cord staining, we used mice not injected with AAV-9.

### Gene expression analysis

Total RNA from cecal tissue was isolated using the RNeasy Mini kit (Qiagen, Valencia, CA). cDNA was synthesized using the PrimeScript 1st strand cDNA Synthesis Kit (Takara Bio, Shiga, Japan). Quantitative reverse transcriptase-PCR was performed using the SYBR Green I Kit (Roche, Mannheim, Germany). We extracted DNA from cecal contents and the spleen using the DNA Stool Mini kit (Qiagen). Quantitative PCR was performed using the SYBR Green or TaqMan methods. The primers used are summarized in Supplementary Table 6. The 16S rRNA gene was used as an internal control in the analysis of the gut bacterium, whereas the 18S rRNA gene was used in the analysis of cecal genes. The comparative ΔCt method was used for both analyses. Analysis of splenic ClpB was performed by quantitative PCR using 100 ng DNA as template. When the amplification curve rose within 40 cycles of the PCR reaction, it was considered positive.

### Norepinephrine measurement

Norepinephrine (NE) levels in plasma and cecal contents were measured using a Noradrenaline Research ELISA kit. We conducted the experiments according to the manufacturer’s protocol. Briefly, after centrifugation at 13,000 × *g* for 10 min, 10 µl of plasma was used. The contents of the cecum were mixed with sterilized water at a rate of 0.25 g per 1 ml and centrifuged at 13,000 × *g* for 15 min, and then 100 µl of supernatant was used. The cross reactivity was 0.14% for adrenaline and 0.2% for dopamine.

### Statistical analysis

Non-paired t-test, one-way ANOVA, two-way ANOVA, and linear regression analysis were performed with GraphPad Prism 7 (GraphPad, San Diego, CA). Data are shown as the mean ± SEM. Statistical significance was set at *P* < 0.05.

## Supplementary Information

Supplementary Information 1.

Supplementary Information 2.

Supplementary Information 3.

Supplementary Information 4.

Supplementary Information 5.

Supplementary Information 6.

## Data Availability

Data are available upon relevant request to the corresponding author.
