# Supplementary figures and images for: Effects of GLP-1 receptor agonist on changes in the gut bacterium and the underlying mechanisms
Source: Sci Rep. 2021 Apr 28;11:9167. doi: 10.1038/s41598-021-88612-x (PMC8080802; doi:10.1038/s41598-021-88612-x)

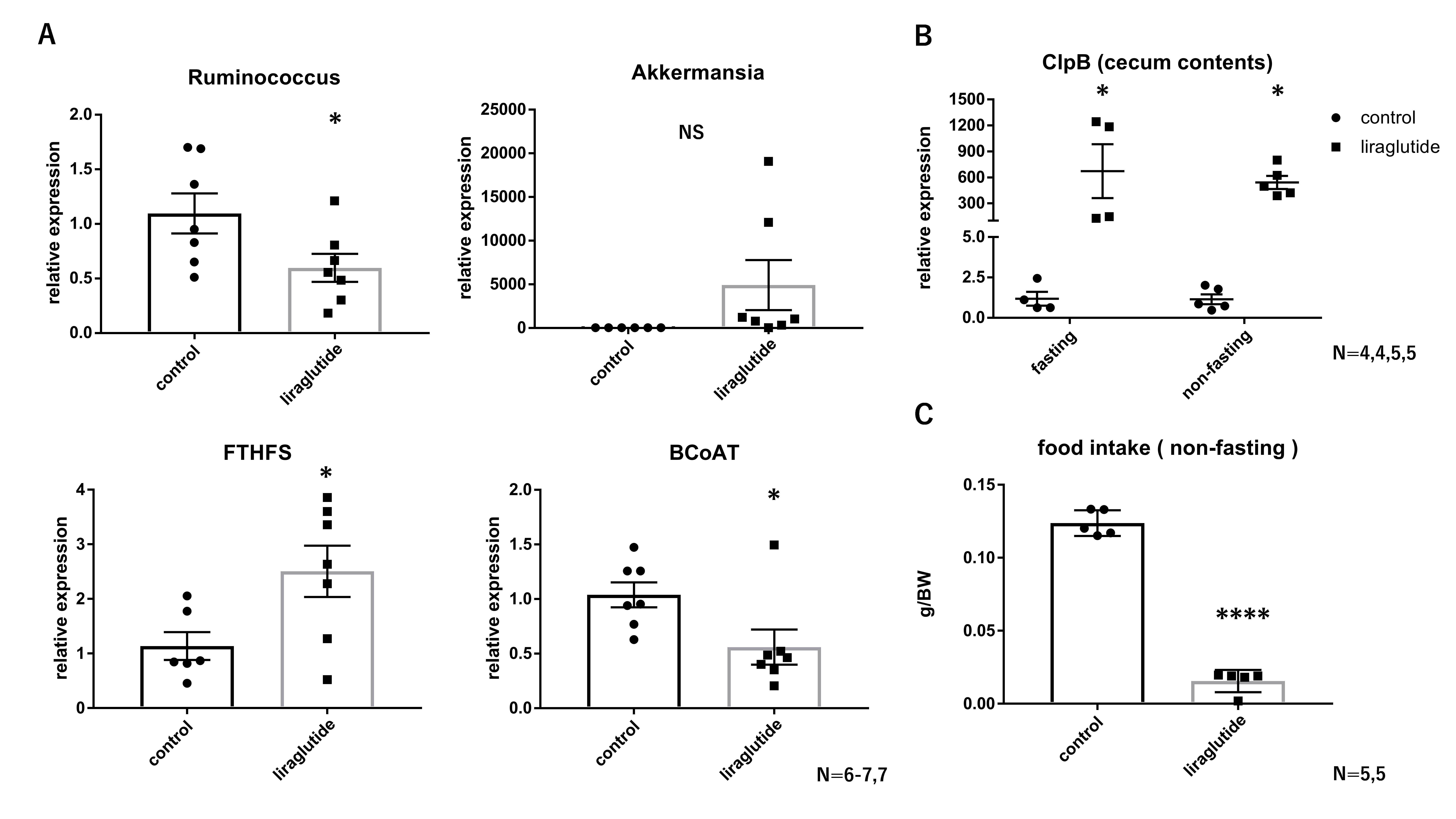

Supplement: Supplementary file 1 — Supplementary Information 1. [file 41598_2021_88612_MOESM1_ESM.tif]

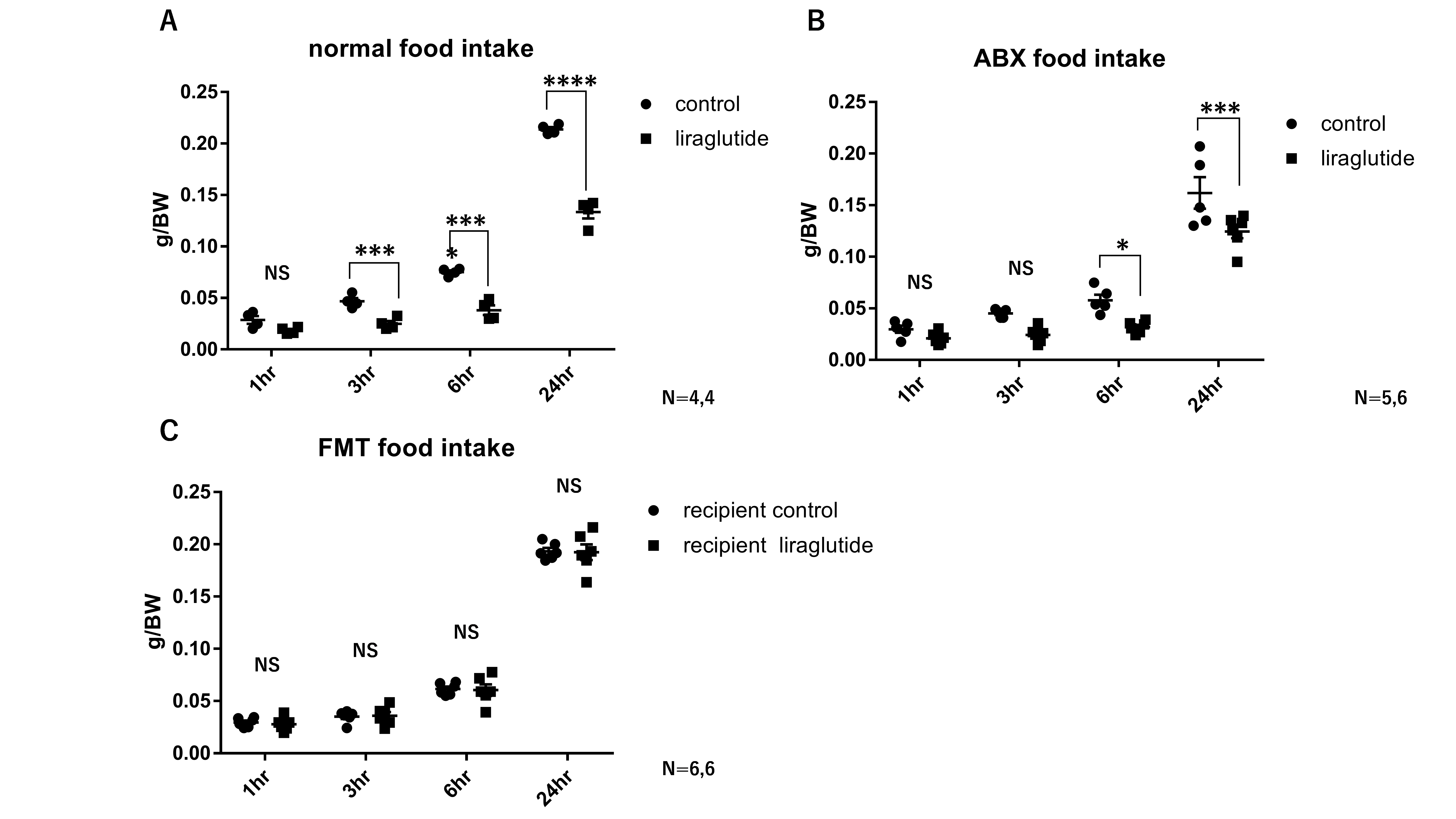

Supplement: Supplementary file 2 — Supplementary Information 2. [file 41598_2021_88612_MOESM2_ESM.tif]

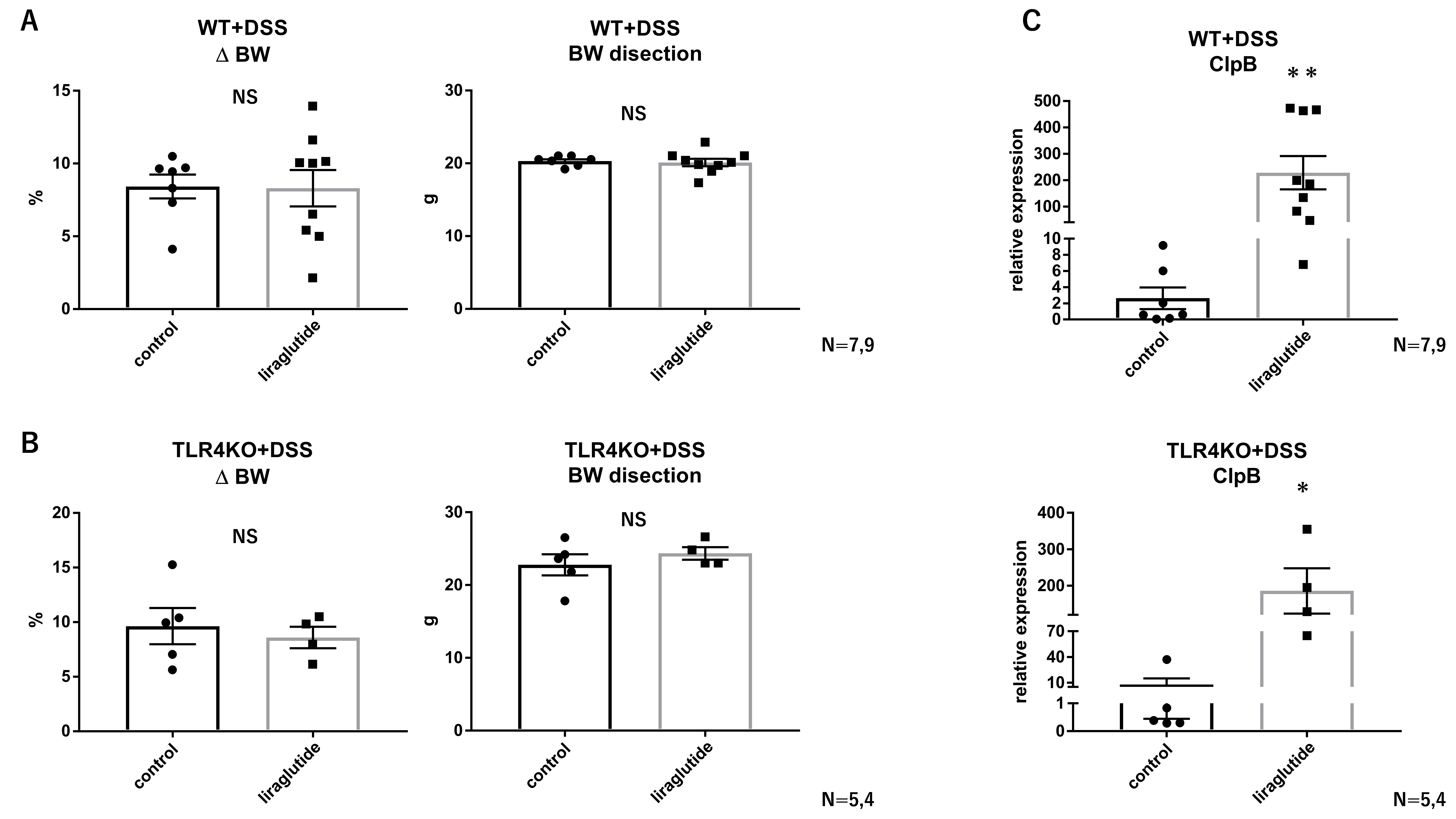

Supplement: Supplementary file 3 — Supplementary Information 3. [file 41598_2021_88612_MOESM3_ESM.tif]

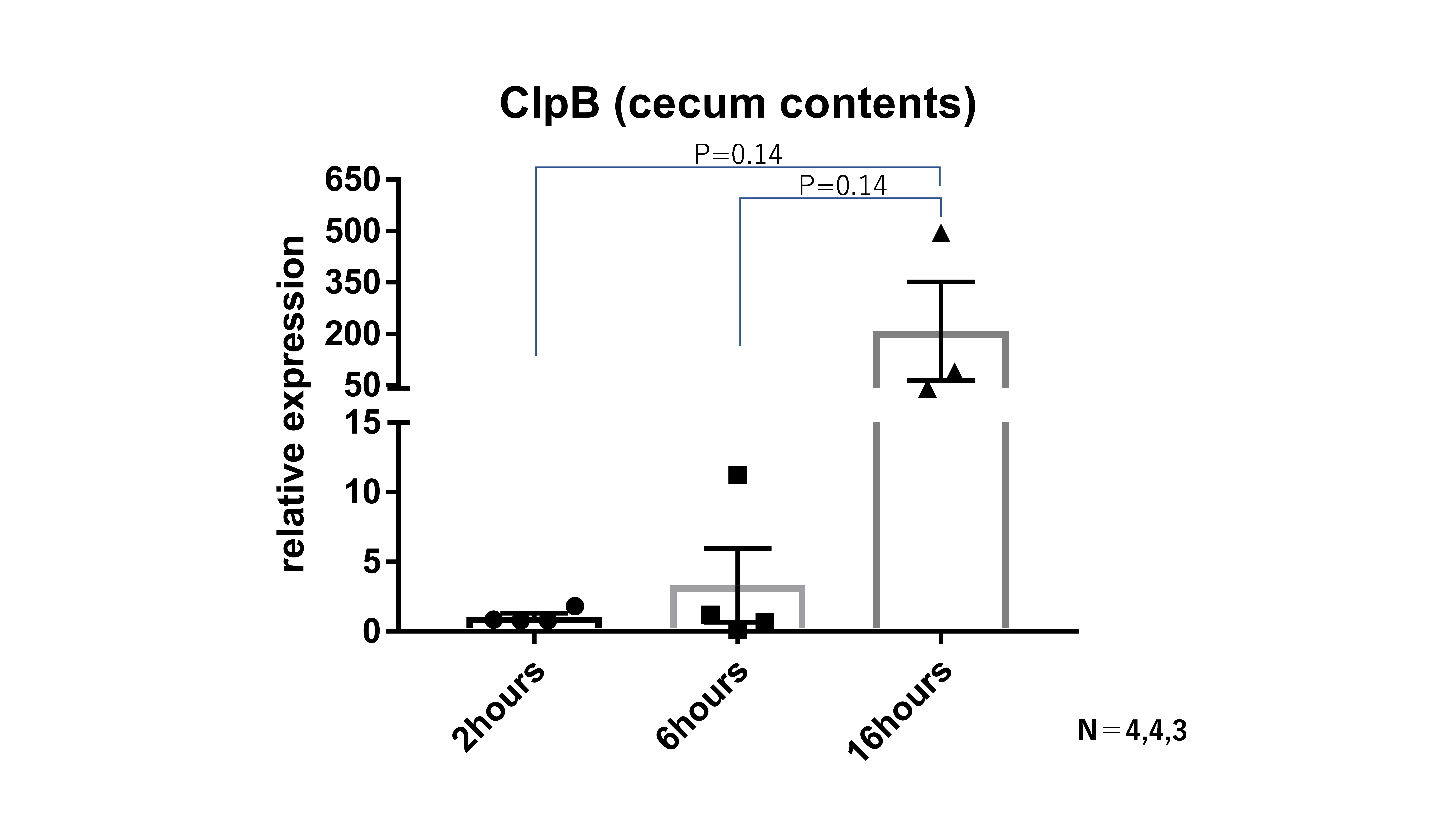

Supplement: Supplementary file 5 — Supplementary Information 5. [file 41598_2021_88612_MOESM5_ESM.tif]
