# Supplementary material for: Effects of GLP-1 receptor agonist on changes in the gut bacterium and the underlying mechanisms
Source: Sci Rep. 2021 Apr 28;11:9167. doi: 10.1038/s41598-021-88612-x (PMC8080802; doi:10.1038/s41598-021-88612-x)
Supplement: Supplementary file 6 — Supplementary Information 6. [file 41598_2021_88612_MOESM6_ESM.docx]

**Supplementary information**

**Title:**

Effects of GLP-1 receptor agonist on changes in the gut bacterium and the underlying mechanisms

Shunsuke Kato^1^, Takehiro Sato^1^, Hiroki Fujita^1^, Masahiro Kawatani^2,3^, Yuichiro Yamada^1,4^

Departments of ^1^Endocrinology, Diabetes, and Geriatric Medicine, and ^2^Neurophysiology, Akita University Graduate School of Medicine, Akita, Japan

^3^Department of Neuroscience II, Research Institute of Environmental Medicine, Nagoya University, Nagoya, Japan

^4^Kansai Electric Power Medical Research Institute, Osaka, Japan

**Supplementary Table 1**

Primer sequences used in this study.

| Target gene | Forward ( 5´-3´) | Reverse ( 5´-3´) |
| --- | --- | --- |
| Universal  (16S rRNA) | ACTCCTACGGGAGGCAGCAGT | ATTACCGCGGCTGCTGGC |
| Firmicutes | GGAGYATGTGGTTTAATTCGAAGCA | AGCTGACGACAACCATGCAC |
| Bacteroidetes | CRAACAGGATTAGATACCCT | GGTAAGGTTCCTCGCGTAT |
| γ-proteobacteria | TCGTCAGCTCGTGTYGTGA | CGTAAGGGCCATGATG |
| Actinobacteria | TACGGCCGCAAGGCTA | TCRTCCCCACCTTCCTCCG |
| ClpB | GCAGCTCGAAGGCAAAACTA | ACCGCTTCGTTCTGACCAAT |
| ClpB (taqman) | GTACTGGCGCTGGATATGG | CTTCAAACTCACCGCGATATT |
| E. coli  (16S rRNA) | CATGCCGCGTGTATGAAGAA | CGGGTAACGTCAATGAGCAAA |
| Ruminococcus | GGCGGCCTACTGGGCTTT | CCAGGTGGATAACTTATTGTGTTA |
| Akkermansia | CAGCACGTGAAGGTGGGGAC | CCTTGCGGTTGGCTTCAGAT |
| FTHFS | GTWTGGGCWAARGGYGGMGAAGG | GTATTGDGTYTTRGCCATACA |
| BCoAT | GCIGAICATTTCACITGGAAYWSITGGCAYATG | CCTGCCTTTGCAATRTCIACRAANGC |
| 18S rRNA | CTCAACACGGGAAACCTCAC | CGCTCCACCAACTAAGAACG |
| occuldin | CACACAGGACATGCCTCCAC | GGCTGCCTGAAGTCATCCAC |
| TNF-α | ACTGAACTTCGGGGTGATTG | GCTTGGTGGTTTGCTACGAC |
| RegIIIβ | TGCCTTAGACCGTGCTTTCT | TCCCTTGTCCATGATGCTCTT |
| IL-33 | TGAGACTCCGTTCTGGCCTC | CTCTTCATGCTTGGTACCCGAT |
| zo-1 | ACAGGCCATTACGAGCCTCT | GGAGGCTGTGGTTTGGTAGC |
| claudin-2 | TATGTTGGTGCCAGCATTGT | TCATGCCCACCACAGAGATA |
| claudin-1 | TTCTGGGAGGTGCCCTACTT | TGGATAGGGCCTTGGTGTTG |
| Tff2 | TGCTCTGGTAGAGGGCGAG | CGACGCTAGAGTCAAAGCAG |
| S100A8 | GCCCTCTACAAGAATGACTTCAAG | ATCACCATCGCAAGGAACTCC |
| Lcn2 | ACATTTGTTCCAAGGTCCAGGGC | CATGGCGAATGGTTGTAGTCCG |
| MUC2 | ACCTGGGGTGACTTCCACT | CCTTGGTGTAGGCATCGTTC |
| MUC3 | AAAGATTACCTCCCATCTCC | TAAAACTAAGCATGCCCTTG |
| MUC5b | ACTTGAGGAGGGTTCCAGGT | ACAGTGCCAGGGTTTATGC |
| IL-18 | GCCTCAAACCTTCCAAATCA | TGGATCCATTTCCTCAAAGG |
| IL-13 | GCAGCATGGTATGGAGTGTG | TGGCGAAACAGTTGCTTTGT |
| IL-17A | CAGACTACCTCAACCGTTCCAC | TCCAGCTTTCCCTCCGCATTGA |
| IL-22 | ATCAGTGCTACCTGATGAAG | CATTCTTCTGGATGTTCTGG |

**Supplementary Figure**

**Supplementary Figure 1**

**
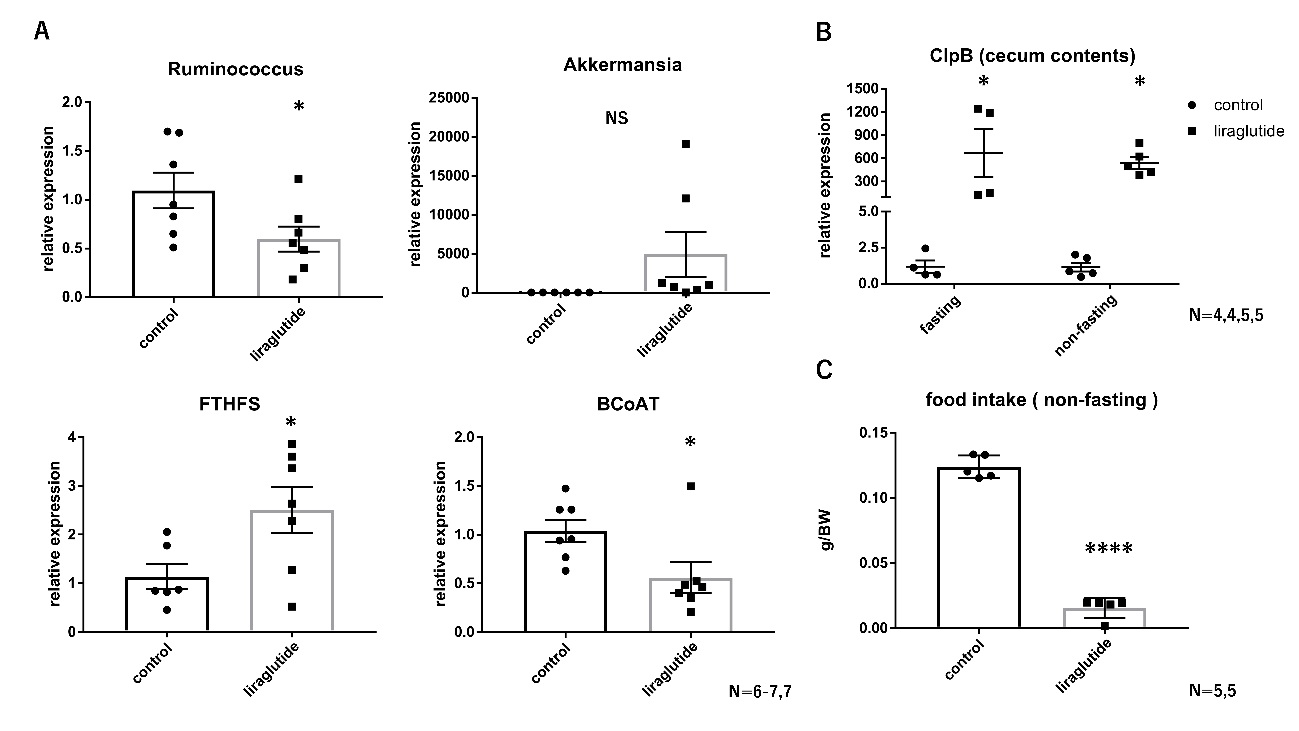
**

**Supplementary Figure 2**

**
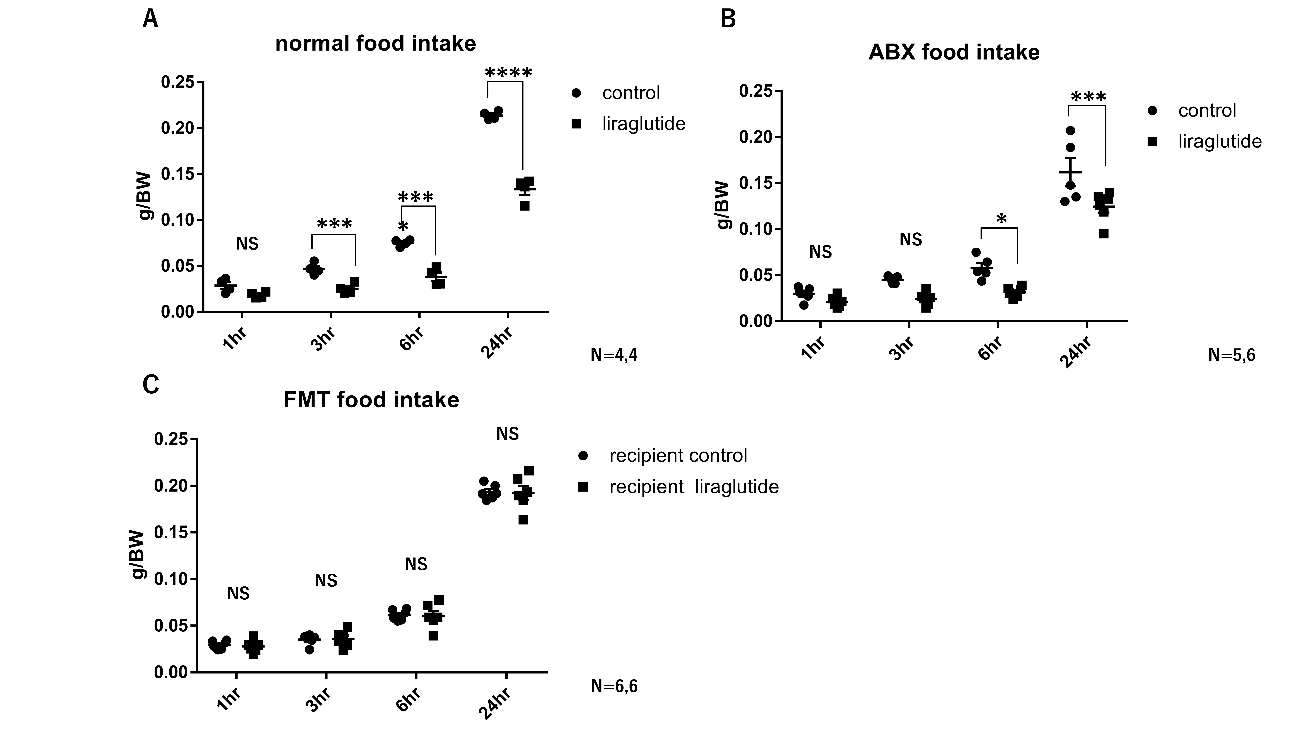
**

**Supplementary Figure 3**

**
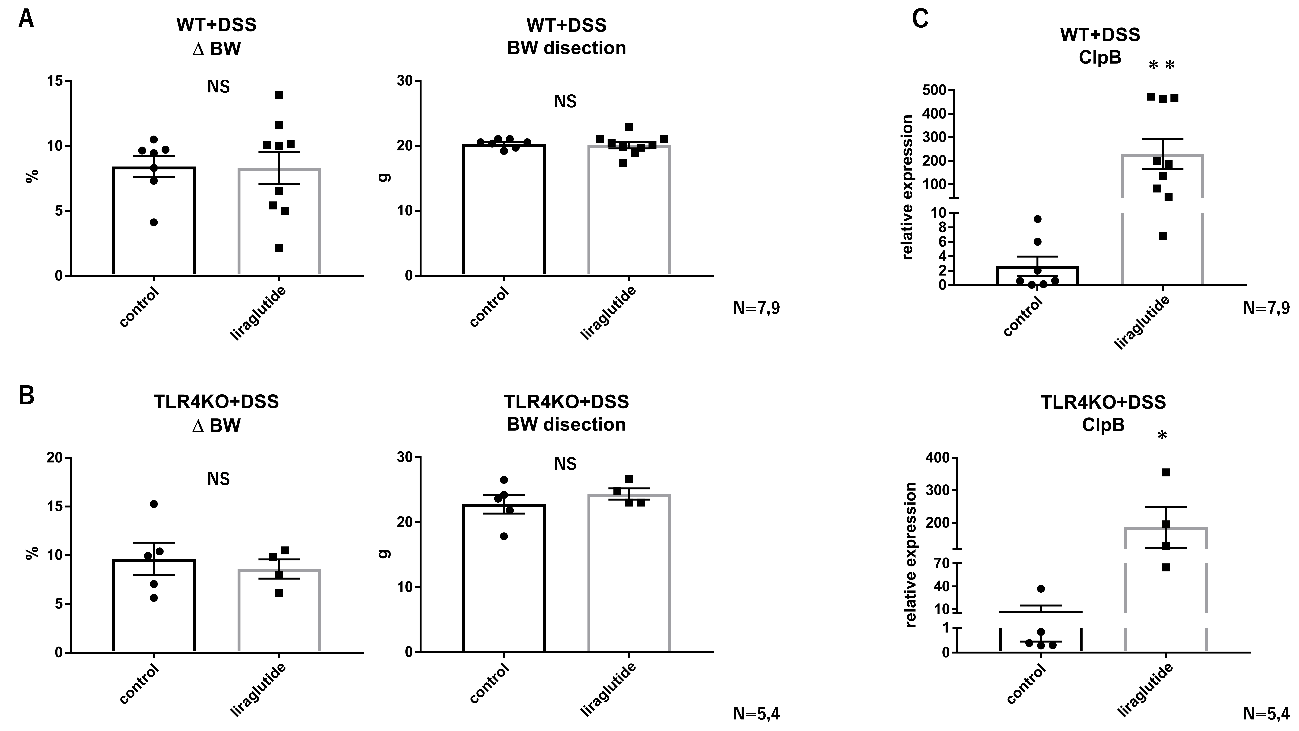
**

**Supplementary Figure 4**

**
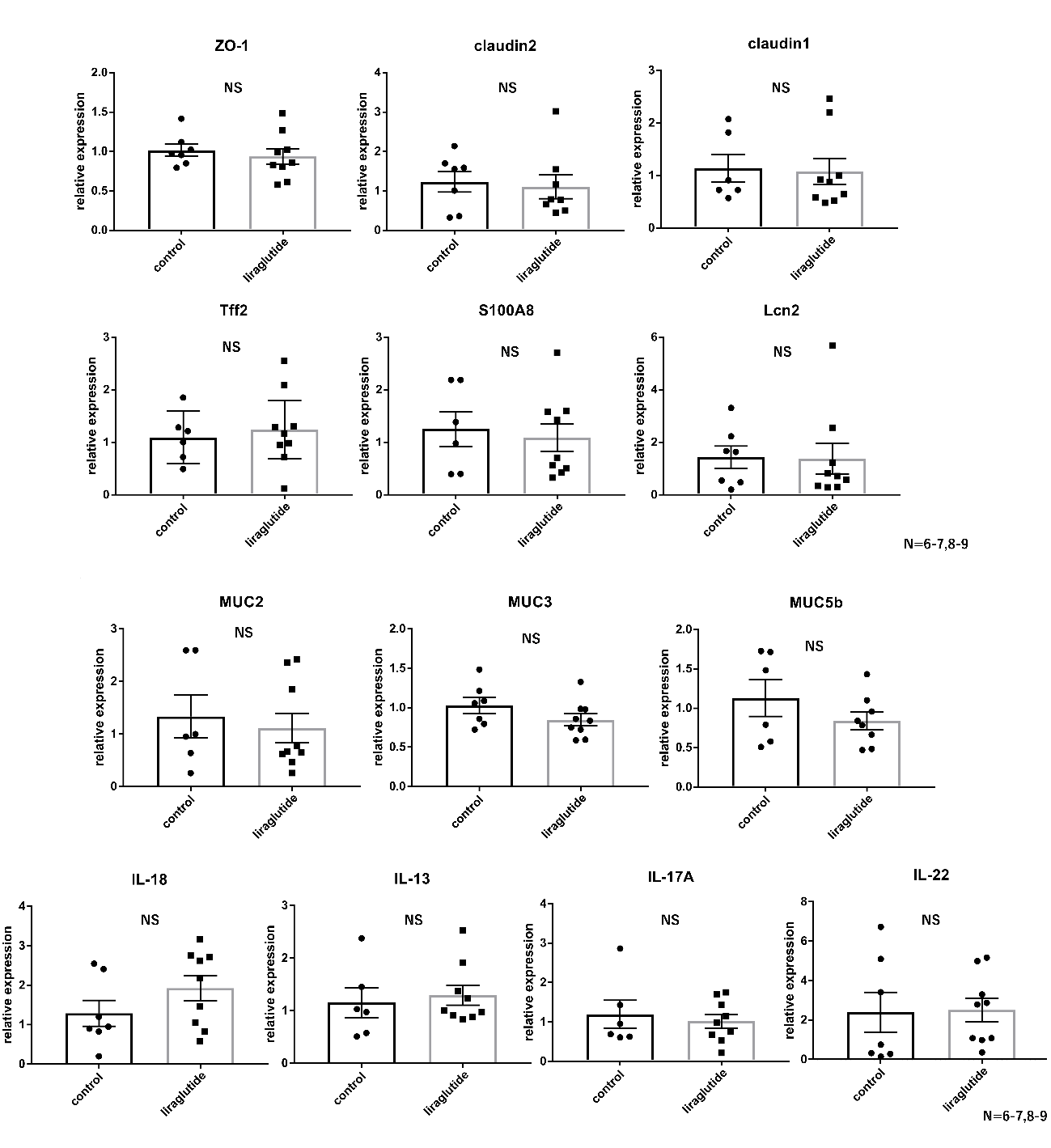
**

**Supplementary Figure 5**

**
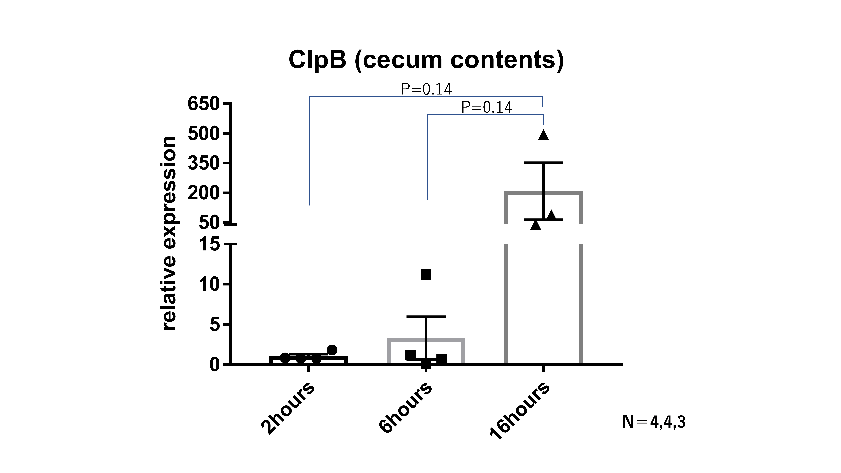
**

**Figure Legends**

**Supplementary Figure 1**

**Liraglutide alters** **the gut bacteria and bacterial genes in the fasting condition, and increases cecal ClpB expression in the non-fasting condition with decreasing food intake.**

(A) Relative expression of the gut microbiota and of bacterial protein-coding genes in cecal contents with or without acute liraglutide administration (control, n = 6–7; liraglutide, n = 7). (B) Relative expression of ClpB in cecal contents in the fasting and non-fasting conditions with or without acute liraglutide administration (fasting-control, n = 4; fasting-liraglutide, n = 4, non-fasting control, n = 5, non-fasting liraglutide, n = 5) (C) Cumulative food intake after 16-hour with or without acute liraglutide administration (control, n = 5; liraglutide, n = 5). All values represent the mean ± SEM. *P < 0.05, **P < 0.01, ***P < 0.001; NS, not significant by non-paired t-test (A and C) and two-way ANOVA with multiple comparison test (B). All figures were drawn using GraphPad Prism 7 (www.graphpad.com) and Microsoft’s PowerPoint 2016 (www.microsoft.com).

**Supplementary Figure 2**

**Increased expression of ClpB does not affect appetite.**

Cumulative food intake after 16-hour fasting with or without acute liraglutide administration (A) in normal wild-type mice (control, n = 4; liraglutide, n = 4) or (B) in antibiotic (ABX)-treated gut microbiota-suppressed mice (control, n = 5; liraglutide, n = 6). (C) Cumulative food intake after 16-hour fasting in ABX-treated gut microbiota-suppressed mice (FMT at the start of food intake measurement; recipient control, n = 6; recipient liraglutide, n = 6). All values represent the mean ± SEM. *P < 0.05, **P < 0.01, ***P < 0.001; NS, not significant by non-paired t-test. All figures were drawn using GraphPad Prism 7 (www.graphpad.com) and Microsoft’s PowerPoint 2016 (www.microsoft.com).

**Supplementary Figure 3**

**In DSS colitis, liraglutide increases cecal ClpB expression in WT and TLR4KO mice, with no body weight difference.**

Rate of weight loss before and after the start of DSS drinking and weight on the day before dissection with or without acute liraglutide administration (A) in wild-type (WT) mice (control, n = 7; liraglutide, n = 9) and (B) TLR4KO mice (control, n = 5; liraglutide, n = 4). (C) Relative expression of ClpB in cecal contents under DSS colitis conditions with or without acute liraglutide administration in wild-type (WT) mice (control, n = 7; liraglutide, n = 9) and TLR4KO mice (control, n = 5; liraglutide, n = 4). All values represent the mean ± SEM. *P < 0.05, **P < 0.01, ***P < 0.001; NS, not significant by non-paired t-test. All figures were drawn using GraphPad Prism 7 (www.graphpad.com) and Microsoft’s PowerPoint 2016 (www.microsoft.com).

**Supplementary Figure 4**

**In DSS colitis, liraglutide does not alter several gene expressions involved in intestinal defense.**

Relative expression of cecal genes with or without liraglutide administration under DSS colitis conditions in wild-type mice (control, n = 6–7; liraglutide, n = 8–9). All values represent the mean ± SEM. *P < 0.05, **P < 0.01, ***P < 0.001; NS, not significant by non-paired t-test. All figures were drawn using GraphPad Prism 7 (www.graphpad.com) and Microsoft’s PowerPoint 2016 ([www.microsoft.com](http://www.microsoft.com)).

**Supplementary Figure 5**

**Comparison of relative expression of cecal ClpB after liraglutide administration**

Relative expression of cecal ClpB at 2, 6, 16 hours after liraglutide administration under fasting conditions in wild-type mice (2 hours, n = 4, 6 hours, n = 4, 16 hours, n = 3). All values represent the mean ± SEM. *P < 0.05, **P < 0.01, ***P < 0.001; NS, not significant by one-way ANOVA with multiple comparison test. All figures were drawn using GraphPad Prism 7 (www.graphpad.com) and Microsoft’s PowerPoint 2016 ([www.microsoft.com](http://www.microsoft.com)).
